# Past and present of cystic echinococcosis in Bolivia

**DOI:** 10.1371/journal.pntd.0009426

**Published:** 2021-06-17

**Authors:** Viterman Ali, Eddy Martinez, Pamela Duran, Erick Villena, Peter Deplazes, Cristian A. Alvarez Rojas

**Affiliations:** 1 Instituto de Investigación en Salud y Desarrollo (IINSAD); Cátedra de Parasitología, Facultad de Medicina, Universidad Mayor de San Andrés (UMSA), La Paz, Bolivia; 2 Programa Regional de Control de Hidatidosis, Red de Salud de Tupiza-Potosí, Bolivia; 3 Institute of Parasitology, Vetsuisse Faculty, University of Zürich, Switzerland; Federation University Australia, AUSTRALIA

## Abstract

Viable eggs of the canine intestinal tapeworm *Echinococcus granulosus sensu lato* (*s*.*l*.) infect various intermediate hosts causing cystic echinococcosis (CE). Furthermore, CE represents a serious zoonosis causing a significant global burden of disease. CE is highly endemic in South America, including Argentina, Brazil, Chile, Uruguay, and Peru. For Bolivia, no official data concerning the incidence in humans or the number of livestock and dogs infected are available. However, it is well known that CE occurs in Bolivia. We aim here to fill the gap in the current knowledge of the epidemiological situation of CE in Bolivia, providing a historical overview of documents published within the country, which have never been comprehensively reviewed. The very first documentation of *E*. *granulosus* infection in animals dates in 1910, while the first human case was reported in 1913. In total, 876 human CE cases have been reported in the scientific literature, with an apparent increase since the 1970s. In the absence of other epidemiological studies, the highest prevalence in human comes from Tupiza, Potosí Department, where 4.1% (51/1,268) of the population showed signs of CE at mass ultrasound screening in 2011. In the same report, 24% of dog faecal samples were positive for coproantigens of *E*. *granulosus s*.*l*. in ELISA. The highest prevalence in intermediate hosts reported at abattoir reached 37.5% in cattle from Potosí, followed by 26.9% in llamas from Oruro, 2.4% in pigs and 1.4% in sheep from La Paz. Finally, *Echinococcus granulosus sensu stricto* (*s*.*s*.), *Echinococcus ortleppi* (G5), and *Echinococcus intermedius* (G7) have been identified in Bolivia. Data reviewed here confirm that *E*. *granulosus s*.*l*. is circulating in Bolivia and that a proper prospective nationwide epidemiological study of CE is urgently needed to define transmission patterns as a basis for the planning and implementation of future control measurements.

## Introduction

Cystic echinococcosis (CE), caused by *Echinococcus granulosus sensu lato* (*s*.*l*), is highly endemic in regions of Argentina, Brazil, Chile, Peru, and Uruguay [1]. Substantial research on different aspects of the epidemiology of CE in these countries has been published. Furthermore, these 5 countries have joined forces since 2004 in “The Regional Initiative for the Control of CE” sponsored by the Pan-American Health Organization (PAHO) [2]. In the case of Bolivia, data regarding any aspect of the epidemiology of CE are not well documented. Moreover, Bolivia is not yet an official member of the international control initiative mentioned above. Reviews of CE in South America have anticipated that the disease must be an important issue in Bolivia. First, Peter Schantz in the 1970s [3,4] highlighted the lack of information related to CE in Bolivia. The only available data (at the time) indicated that 75 CE surgeries were performed in a hospital from the Altiplano region in 10 years. Neghme [5] also suggested (in 1987) that CE was present in Bolivia, but it was less common than in other South American countries. By 1995, it was believed that CE was endemic in the mountainous regions of Bolivia [6]. Moro and Schantz [7] published, in 2006, the latest revision of the situation of CE in the Americas without adding new data due to the apparent lack of original publications from Bolivia. The most up-to-date map of the global distribution of *Echinococcus* spp. suggests that the disease has extended to La Paz, Cochabamba, and Potosí [1]. Recently, molecular characterisation of *E*. *granulosus s*.*l*. in different hosts in Bolivia identified *Echinococcus granulosus sensu stricto* (*s*.*s*.), *Echinococcus ortleppi*, and *Echinococcus intermedius* (G7) [8].

Bolivia has 10.9 million inhabitants from 36 ethnicities and 37 official languages, with 41% of the population identifying as indigenous [9,10]. Bolivia remains the poorest country in South America, with a gross national income of 6,290 USD per capita [11]. A reduction in the number of people living under extreme poverty has been recently observed from 37% in 2007 to 17.3% in 2014 [11]. Several infectious diseases, many conventionally called neglected, are endemic in different parts of Bolivia including tuberculosis [12], malaria [13], Chagas [14], leprosy [15], leishmaniosis [16], soil-transmitted helminths [17,18], fasciolosis [19], rabies [20], and emerging viral pathogens like dengue [21], chikungunya [22], and zika [23]. With such a plethora of important pathogens, it is not surprising that CE, which is a typically chronic disease (frequently asymptomatic), has received little attention in Bolivia. The absence of an official collection of data for human and animal infection has precluded the accurate understanding of CE’s epidemiology in this country. However, several documents showing data of *E*. *granulosus s*.*l*. in humans and animals in Bolivia have been published since 1910; these include undergraduate and postgraduate theses, case reports, retrospective studies, books, and government documents. Most of these documents are not available online and remain in local university libraries, institutional archives, or private collections. We aim to deliver a historical overview of such data providing an initial step for further epidemiological investigations of CE in Bolivia.

## Methods

A literature search was conducted between 2015 and 2020. Scientific databases including PubMed, Web of Science, Scielo, and PAHO were consulted. We used combinations of the following keywords: Bolivia, *Echinococcus granulosus*, *Echinococcus*, cystic echinococcosis, hydatidosis, hidatidosis, hydatid disease, and *Equinococus*. However, most of the documents used in this review were acquired by physically visiting different university libraries in Bolivia. These included the Faculty of Medicine, Faculty of Pharmaceutical and Biochemical Sciences, and the Faculty of Agronomy at the Universidad Mayor de San Andrés (UMSA), La Paz; the “Centro de Información y Gestión de Conocimiento OPS/OMS-UMSA,” La Paz; the Faculty of Veterinary Medicine at the Universidad Gabriel René Moreno, Santa Cruz; the Universidad Mayor Real y Pontificia San Francisco Xavier de Chuquisaca; and the Municipal Library of La Paz. Articles (and grey literature) in English or mainly in Spanish, which were not available in the sources mentioned, were retrieved from particular libraries (or collections), including one of the most important private collections of medical literature in Bolivia owned by Dr Rolando Costa Arduz.

### Cystic echinococcosis in humans

#### Reporting of CE cases in Bolivia

**Fig 1:**
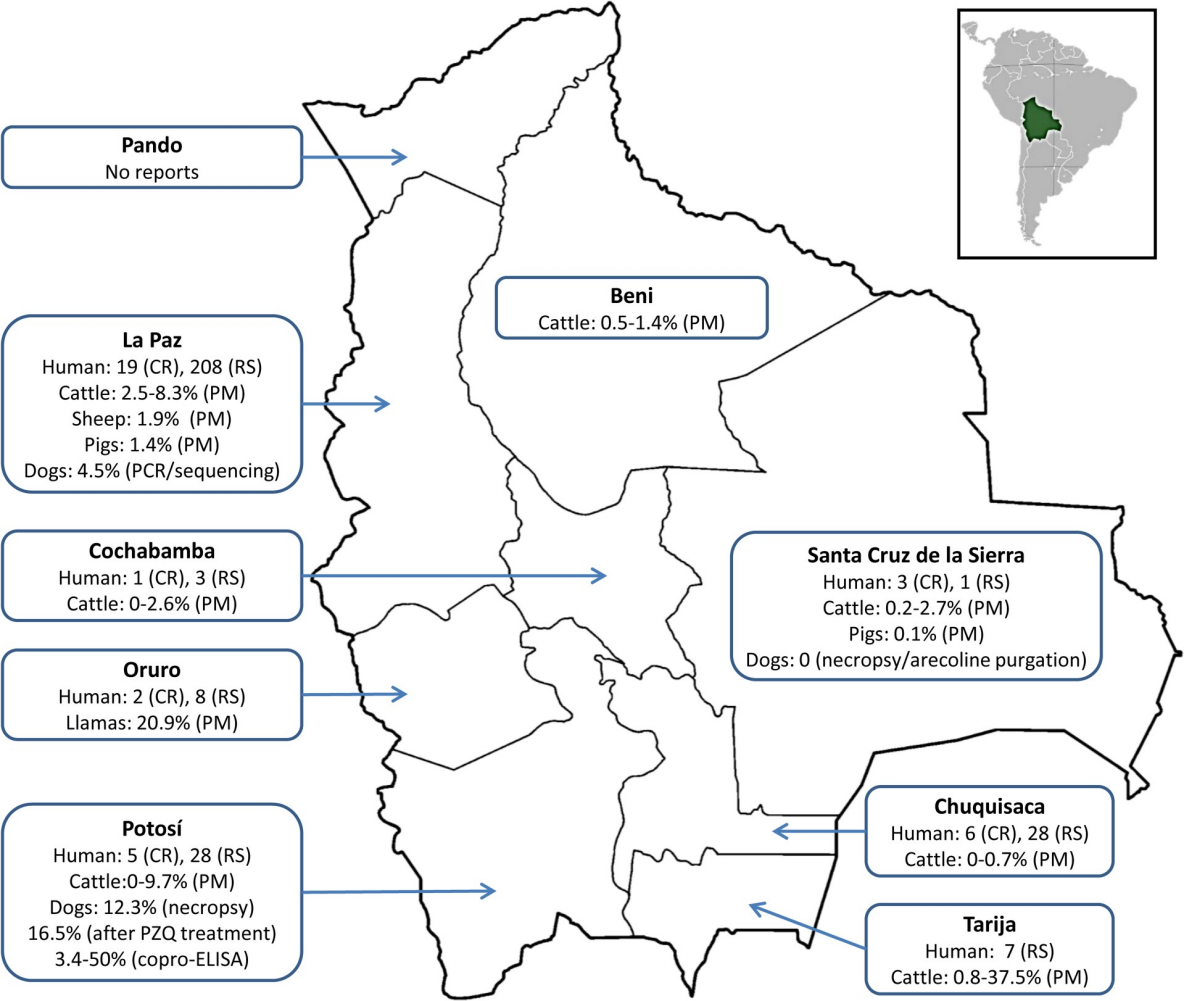
Distribution of human CE cases in Bolivia reviewed in this study from case reports and retrospective studies; maximum and minimum prevalence recorded in livestock at official postmortem examination at abattoir; and in dogs established at necropsy, arecoline purgation, after treatment with praziquantel, copro-ELISA, and PCR/sequencing. In total, there are 38 human case reports (for 2 cases, the origin is unknown) and 517 cases from retrospective studies; only 284 cases were included in the figure due to the lack of information about the origin of the patients. Original base layer of the map can be found here: https://commons.wikimedia.org/wiki/File:Bolivia_departments_blank.png. CR, case report; PM, postmortem; PZQ, praziquantel; RS, retrospective study.

Table 1 summarises the 38 published CE case reports of Bolivian patients, including 2 individuals who lived shortly in the country and were later diagnosed and treated in the United States and Brazil [24,25]. The distribution of case reports in the different Bolivia Departments for which the patients’ origin was known is shown in Fig 1. The oldest mention of a CE case, which we were able to find in the literature, appeared in a book published in 1956, which summarises the history of Medicine in Bolivia [26]; in this book, the author reports CE occurring in 1913 in the liver and lung of patients from the Department of Sucre. While the first CE case report described in 1928 detailed information of CE affecting the pectoral muscle of an 18-year-old patient from the Sucre Department [27]. Interestingly, this author suggests that the parasite is rare in autopsies and livestock animals in this city. However, 3 other cases from Sucre are mentioned in the discussion of the same report affecting the liver, brain (subsequently published by Fernandez [28]), and the orbital cavity (later published by Solares [29]). The first paediatric case was reported in 1965 by Daza and colleagues [30], describing a 15-cm cyst located in the spleen of an 11-year-old female from La Paz. The youngest case published reported CE in a 3-year-old female patient who lived shortly in La Paz before moving to Sao Paulo (Brazil), where she was diagnosed and treated [25]. Paediatric cases are of particular importance since diagnosis in children is a sign of the parasite’s active recent transmission. Simultaneous CE and tuberculosis (TBC) infection have been reported [31], TBC can also complicate the proper diagnosis of pulmonary CE [32]. This is relevant since TBC is highly endemic in some areas of Bolivia. In 2012, a CE case reached national relevance in the media when a cyst of 15 kilograms was diagnosed in a 25-year-old female from La Paz; a video with a synopsis of the surgical procedure can be found on the internet [33]. In the discussion of some of the case reports summarised in Table 1, there are hints of the relevance and awareness of CE’s existence in Bolivia, showing how the perception of the disease’s presence changed over time. Solares [34], in 1948, suggested that CE was not uncommon in Bolivia. Differently, Boehme [35], in 1953, considered CE as a sporadic disease in Bolivia. Daza, in 1965, mentioned that CE cases became commonly diagnosed in recent years [30]. In 2005, Aguirre [36] noted that according to a study of the Bolivian National Statistical Institute, there were 500 new CE cases, with a mortality rate between 12% and 15%. Unfortunately, we could not have access to such a document. Vera and colleagues [37], in 2006, mentioned that CE has gone from a rare finding to become endemic due to the significant increase in lung cases in La Paz. Burgos-Burgoa also recognised that CE in the lungs had become also frequently diagnosed in Cochabamba in recent years [38].

**Table 1:** Chronological overview of case reports of human CE patients from Bolivia between 1913 and 2020.

Year	Department	Gender	Age (years)	Localisation	Basis for defining a CE case	Laboratory confirmation	Comments	Reference
1913	Chuquisaca	ND	ND	Lungs/Liver	ND	ND	-	[26]
1928	Chuquisaca	Male	18	Muscle	Surgery	Microscopy	Clear fluid at the puncture	[27]
		ND	ND	Liver	Surgery	ND	-	
		ND	ND	Orbital cavity	Surgery	ND	-	
1929	Chuquisaca	Male	58	Brain	Autopsy	Microscopy	Postmortem	[28]
1932	Potosí	Female	47	Orbit	Surgery	Microscopy	Surgically intervened	[29]
1948	Unknown	Unknown	Unknown	Orbit	Surgery	ND	Two different cases	[34]
	Unknown	Unknown	Unknown	Orbit	Surgery	ND		
1953	La Paz	Male	33	Bile ducts	Surgery	Microscopy	Polish patient lived in Argentina	[35]
		ND	ND	Lungs	Surgery	ND	-	
		ND	ND	Spinal cord	Surgery	ND	-	
1965	La Paz	Female	11	Spleen	X-ray, surgery	Microscopy and histopathology	First paediatric case	[30]
1969	Oruro	Male	56	Mediastinum	X-ray, surgery	Microscopy and histopathology	Initially diagnosed as lung cancer	[39]
1969	Santa Cruz	ND ND	ND ND	Lungs Lungs	X-ray X-ray	ND ND		[40]
1971	Oruro	Female	20	Lungs	X-ray, surgery	ND	Simultaneous TBC	[31]
1973	Santa Cruz	Male	5	Lungs	X-ray	ND	25 cm diameter	[41]
1987	US citizen who lived shortly in La Paz	Female	16	Lungs	X-ray, ultrasound, CT scan, surgery	Microscopy and histopathology	Surgically intervened in the USA	[24]
1995	Potosí	Female	NA	Spine	Surgery	Histopathology	Surgically intervened	[42]
2004	La Paz	Male	17	Lungs	X-ray, ELISA	No	Initially diagnosed with TBC. Treated with mebendazol	[32]
2004	Chuquisaca	Female	18	Lungs	X-ray, CT scan, ELISA (-), surgery	ND	Four cysts surgically intervened	[43]
2005	La Paz	Male	54	Liver and peritoneum	Surgery	Microscopy and histopathology	Lives with 4 dogs	[44]
2005	La Paz	Male	13	Lungs	X-ray, vomica	ND	Treated with albendazole	[45]
2005	La Paz	Female	19	Abdomen	Ultrasound, surgery	Microscopy and histopathology	Cyst measuring 80 cm in a pregnant woman (24 weeks)	[36]
2005	La Paz	Female	37	Spleen	Ultrasound, surgery	Histopathology	Patient operated years before for pulmonary cysts	[46]
2006	La Paz	Male	19	Lungs	X-ray, surgery	ND	Cyst measuring 15 × 25 cm	[37]
	La Paz	Male	34		X-ray, surgery	ND	Cyst measuring 10 cm	
	La Paz	Male	23		X-ray, surgery	ND	Cyst measuring 20 cm	
2009	La Paz	Male	29	Liver	Ultrasound, serology, surgery	Histopathology	Cyst measuring 16 cm. Surgically treated	[47]
2009	Potosí (Villazón)	Male	47	Liver	Ultrasound, ELISA (-), surgery	Microscopy and histopathology	Cyst measuring 16 cm	[48]
2012	La Paz	Female	25	Abdomen	Ultrasound, surgery	Microscopy and histopathology	A primary giant cyst (15 kilograms) from a pregnant woman. Pregnancy ended successfully	[33]
2013	Cochabamba	ND	82	Lungs	Ultrasound, CT scan, surgery	Microscopy and histopathology	Two cysts of 11 and 13 cm	[49]
2014	Potosí	Female	33	Lungs	X-ray, ELISA, surgery	Histopathology	A pregnant patient who has contact with dogs	[38]
2014	La Paz	Female	23	Retroperitoneum	CT scan, surgery	Histopathology	-	[50]
2015	Potosí (treated in Cochabamba)	Male	34	Lungs	X-ray, ultrasound, western blot	No	Multiple lung cysts and one in the liver treated albendazole	[51]
2016	La Paz	Female	33	Lungs	X-ray, ELISA, agglutination test	No	Has contact with dogs and sheep	[52]
2018	La Paz	Female	72	Liver	Endoscopy	No	Acute cholangitis	[53]
2020	La Paz (treated in Brazil)	Female	3	Liver	Surgical, X-ray, CT scan	PCR	Born in a rural area near city of La Paz but lives in Brazil, 3 cysts of 9.3–10 cm	[25]

CE, cystic echinococcosis; ND, no data; TBC, tuberculosis.

#### Retrospective studies

To date, there are 8 published retrospective studies regarding CE in Bolivia [54–61], which, in total, account for 518 CE human cases (Table 2). Fig 1 shows the distribution of cases from retrospective studies in Bolivia’s different Departments for which the patients’ origin was known. The first retrospective study of CE is a thesis from the Universidad Mayor Real y Pontificia de San Francisco Xavier in Chuquisaca. CE was identified in the report of 29 out of 2,980 autopsies performed between 1940 and 1954 in the Department of Chuquisaca [60]. Moreno [56] reported 64 CE cases out of 948 thorax surgeries performed between 1968 and 1977 in 1 hospital from La Paz. Interestingly, Moreno and colleagues [56] described that a high proportion of the 64 CE cases lived in mining or urban areas and also highlighted the high number of dogs present in Bolivia at the time [56]. Different authors of retrospective studies suggested that the disease became a significant public health issue and was likely to be underdiagnosed [56,59,60]. An increase in number was also observed in paediatric surgical cases in infants from rural areas living in the peri-urban areas of La Paz and El Alto [58]. More recently, Rodriguez-Andrade and colleagues [61] reported 17 paediatric cases (under 15 years old) between 2015 and 2017 from the intensive care unit of the Children Hospital in La Paz. Only a single publication of the retrospective studies (Table 2) suggested that CE is not an essential public health issue after reporting only 9 hepatic cases between January 1996 and May 2008 from all abdominal surgeries performed at a hospital in La Paz [54]. The majority of the cases visited in these retrospectives studies originated in La Paz, followed by Potosí [57,59]. In the case of the retrospective study by Guzman [55], many patients diagnosed in Tupiza (Potosí) had relatives who previously received surgical treatment for CE. The latter agrees with the high prevalence of humans in Tupiza (4.1%) in 2011 [62]. The other 4 publications mentioned several CE cases without supplying detailed information (not included in Table 2). First, Perez Fontana [63] indicated that Bolivia’s representative in a meeting of PAHO reported 64 CE cases; some of these cases were diagnosed in Santa Cruz. Maldonado [31] mentioned in the discussion of a case report that 83 CE cases without concomitant TBC were surgically treated at the Thorax National Institute without supplying a time frame in which these cases were reported. Finally, in 2 reports for PAHO [3,4], Schantz mentioned that 75 surgeries were due to CE out of 1,500 thorax surgeries in 10 years in La Paz.

**Table 2:** Chronological summary of retrospective studies of human CE reported in Bolivia by department.

Period studied	Department (Hospital/University)	# of cases	Basis for defining a CE case	Comments	Reference
1940–1954	Chuquisaca, (Instituto de Patología, Facultad de Medicina)	29	Autopsy and histopathology	2,980 autopsies. Origin of the patients: 24 from Chuquisaca (82.8%), 3 from Potosí (10.3%), 1 from Santa Cruz (3,4%), and 1 from Oruro (3.4%)	[60]
1968–1977	La Paz (Servicio de cirugía de la Caja y Hospital COMIBOL)	64	Histopathology after surgery	These 64/75 were confirmed CE cases. All cysts located in the lungs. One patient presented vomica.	[56]
1991–1996	La Paz, Three hospitals: Instituto Nacional del Tórax, Luis Uria de la Oliva and Instituto de Gastroenterologia Boliviano Japones plus Instituto Nacional de Laboratorios de Salud.	148	X-ray for 86 thoracic cysts Ultrasound for 6 hepatic cases Histopathology after surgery for 92 cases DD5* for 56 patients	Origin of the patients: 112 from La Paz (75.7%), 17 from Potosí (11.5%), 7 from Tarija (4.7%), 5 from Oruro (3.4%), 4 from Chuquisaca (2.7%), and 3 from Cochabamba (2%)	[57]
1995–2001	La Paz (Hospital Obrero N° 1, and Luis Uría de la Oliva)	113	X-ray and tomography not in all cases. Histopathology after surgery	71% with unique cysts in lungs and 36.1% with cysts in the liver. Several patients were from Tupiza (Potosí)	[55]
1984–1999	La Paz, (Hospital del Niño)	31	X-ray, DD5* for 11 cases. Histopathology after surgery	Paediatric cases: 25 patients presented cyst in lungs, 10 in the liver, and 9 in other organs. The majority of cases were patients from La Paz and El Alto. In 5 patients, the rupture of a cyst was the cause of hospitalisation, one of which died due to anaphylaxis.	[58]
1998–2004	La Paz, (Instituto Nacional del Tórax)	106	Histopathology after surgery	Cysts located in the lungs and liver. Origin of cases: 96 (90%) from La Paz, 8 (7%) from Potosí, and 2 (3%) from Oruro.	[59]
1996–2008	La Paz (Hospital La Paz)	9	Ultrasound, CT scan. Histopathology after surgery	All hepatic cases. No data regarding the origin of patients.	[54]**
2015–2017	La Paz (Hospital del Niño)	17	ND	All paediatric cases	[61]
**Total cases**		**517**			

*DD5, double diffusion arc 5.

**There is a lack of coherence between the period of study and the date of publication.

CE, cystic echinococcosis; ND, no data.

#### Ultrasound diagnosis, treatment, and patient follow-up

The most accurate and recent data related to human CE cases in Bolivia are found in a government report that showed the results of an ultrasound mass screening performed in Tupiza (Potosí Department). In total, 52 out of 1,268 individuals (4.1%) showed CE’s ultrasound signs with active cysts in all patients. Additional thoracic X-ray showed CE in the lungs of 5 of the same patients with abdominal CE [62]. Patients were treated with albendazole 10 to 15 mg/kg/day for 4 months, and 92% completed the treatment. The follow-up showed that 60% of cysts were classified as transitional at the end of the first month after treatment. By the end of the therapy, 90% of the cysts were classified as inactive [64].

#### Immunological testing of the Bolivian population

We found only a few comments related to immunodiagnostic tests in humans in the literature. Schantz [3,4] briefly mentioned high reactivity in an intradermic test (with an antigen from *E*. *granulosus*) in employees of an abattoir in the Bolivian Altiplano. Commercial [65] and in-house ELISA [66] tests have been used to assess sensitivity and specificity using sera from confirmed CE cases from La Paz and Cochabamba and patients with other parasitic diseases. However, there was no attempt to perform a prevalence study in the general population. As part of the ultrasound study in Tupiza (mentioned above), ELISA and immunochromatography tests (Vircell) were used to detect antibodies against *E*. *granulosus* in the sera of 52 patients diagnosed with abdominal CE. In total, 96.7% of the ultrasound cases were positive in both ELISA and immunochromatography. Finally, a study carried out at the Parasitology, Tropical Medicine, and Environment Unit of the Instituto de Investigación en Salud y Desarrollo (IINSAD-UMSA) determined a CE prevalence of 6.3% (44/696) in population from the northern highland at the Department of La Paz using 2 commercial ELISA kits (Nova Tec Inmunodiagnostica and RIDASCREEN) [67].

### *E*. *granulosus* infection in dogs

Data regarding prevalence in dogs in Bolivia are scarce; all reports of dog infection are summarised in Table 3. Fig 1 shows the Departments of Bolivia for which studies detecting *E*. *granulosus* in faeces have been performed. The oldest report of *E*. *granulosus* infection in dogs dates from 1973 Schantz [7], where 50% of dogs from La Paz were found infected after arecoline purgation. Methodologies to correctly identify *E*. *granulosus* have been used only in 5 publications: Barba [68] did not find positives in the necropsy of 100 dogs from Santa Cruz; Perez [69] did not find infections in 60 dogs purged with arecoline; Villena [70] found adult specimens of *E*. *granulosus* in the faeces of 14 out of 85 dogs (16.5%) treated with praziquantel in Tupiza; Subieta [71] determined a prevalence of 12.3% (45/367) in dogs from Tupiza by necropsy; and finally, Ali and colleagues [8] described 4.5% (6/131) of the dogs, from the Altiplano of La Paz, to be infected with *E*. *granulosus s*.*s*. or *E*. *ortleppi* based on PCR and sequencing. Other reports showed results based on coproantigen detection in faeces [62,72], a method known to produce cross-reactions with *Taenia* species, or microscopic detection of taeniid eggs [73–75], a technique that does not allow differentiation between *Echinococcus* and *Taenia* spp. From the reports using a coproantigen ELISA, Villena determined 24% (63/264) of dog faecal samples as positive with ratios varying between 15% and 62% in different localities of Tupiza (at the border with Argentina) [62]. Subsequently, also using coproantigen ELISA, Casas and colleagues [72] described 3.4% of dogs positive in urban samples from Villazón. In comparison, 30% were positives in the samples taken from a rural area (Lampaya) near the border with Argentina. On the other hand, using microscopic detection of taeniid eggs, Salinas [73] reported 1% (1/100) of dog samples as positive in La Paz. In comparison, Traverso [74] reported 13% of 100 dog faeces positive to taeniid eggs in dogs from Guaqui in La Paz (near the Titicaca lake) and 20.4% (29/142) in dogs from Chucuito at the Titicaca lake in Peru. Gonzales [75] reported 9.1% (31/340) of canine faecal sample infected with taeniid eggs in Tupiza.

**Table 3:** Summary of reports of canine echinococcosis and taeniid infection in Bolivia by departments, municipalities, and towns.

Department/Municipality	Town	# dogs studied	# positives (%)	Diagnostic method	Comment	Reference
La Paz/ND	ND	100	1 (1)	Sedimentation/microscopy*	1998 (positive case Munaypata)	[73]
La Paz/Guaqui	Guaqui	100	13 (13)	Sedimentation/microscopy*	Faeces from owned dogs	[74]
La Paz/Batallas La Paz/Pucarani La Paz/Pucarani La Paz/Tiahuanacu	Batallas Pucarani Lacaya Tiahuanacu **Total**	39 22 22 48 **131**	2 (5.1) 1 (4.6) 1 (4.6) 2 (4.2) **6 (4.5)**	Sedimentation/PCR/sequencing	Environmental faecal samples (*E*. *granulosus s*.*s*. was identified in each of the towns, *E*. *ortleppi* in Batallas and Tiahuanacu)	[8]
Potosí/Tupiza	Tupiza City	340	31 (9.1)	Sedimentation/microscopy*	1998–1999	[75]
Potosí/Tupiza	Salo	8	5 (62.5)	Copro-ELISA	2010–2011	[62]
	Peña Amarilla	19	10 (52.6)			
	Talina	5	2 (40)			
	Palala	14	5 (35.7)			
	Rio Blanco	17	6 (35.3)			
	Santa Rosa	15	4 (26.7)			
	Tambo/Mochara	12	3 (25)			
	Yurcuma	18	4 (22.2)			
	Tapaxa	20	4 (20)			
	Charaja	15	3 (20)			
	Iriccina	6	1 (16.7)			
	Tocloca	13	2 (15.4)			
	Villa Pacheco	20	3 (15)			
	Aguadita	22	1 (4.5)			
	San Gerardo	30	0			
	San Antonio	10	0			
	**Total**	**244**	**53 (21.7)**			
Potosí/Cotagaita	Ramadas	10	5 (50)	Copro-ELISA	2010–2011	[62]
Potosí/Villazón	Chipihuayco	10	5 (50)			
Potosí/Villazón	ND	59	2 (3.4)	Copro-ELISA and copro-western blot	Dog population in Villazón 13,637	[72]
Potosí/Lampaya	ND	10	3 (30)			
Potosi/Tupiza	Yurcuma	10	0	Direct observation of adult worms in faeces	Samples collected after praziquantel treatment	[70]
	Villa Pacheco	9	2 (22.2)			
	Tocloca	10	0			
	Tapaxana	9	3 (33.3)			
	Talina	5	1 (20)			
	Santa Rosa	10	1 (10)			
	San Miguel	10	2 (20)			
	Salo	7	5 (71.4)			
	Iriccina	5	0			
	Charaja	10	0			
	**Total**	**85**	**14 (16.5)**			
Potosí/Tupiza	San Antonio	131	15 (11.5)	Necropsy and detection of adult parasites		[71]
	San Gerardo	47	10 (21.3)			
	Sud	76	8 (10.5)			
	Central	79	5 (6.3)			
	Villa Fátima	34	7 (20.6)			
	**Total**	**367**	**45 (12.3)**			
Santa Cruz/Santa Cruz de la Sierra	Santa Cruz de la Sierra	100	0	Necropsy		[68]
Santa Cruz/Samaipata	Samaipata	60	0	Arecoline purgation		[69]

ND, no data.

*Taeniid eggs detected at microscopic examination were mistakenly considered to be from *E*. *granulosus*.

### Cystic echinococcosis in livestock

No official documents from the government or scientific papers reporting CE in animals from Bolivia at a national level have been published. Table 4 summarises all the reports of CE in animals reviewed by us. Fig 1 shows the distribution of all the studies and their prevalences for different livestock species performed in Bolivia. The first mention of CE in animals from Bolivia is dated in 1910, in a book about the parasites found in the Bolivian Altiplano. Surprisingly, the disease was described in a horse and a donkey [76]. For this review, most data regarding CE in animals were acquired from theses, including 9 from the Faculty of Veterinary Medicine at the University Gabriel René Moreno in Santa Cruz de la Sierra; 1 thesis from the Universidad de El Alto, 1 from the Faculty of Agronomy at UMSA, and 1 from the Faculty of Veterinary Medicine at the University Tomas Frias (Table 4). A study in 1969 reported 31 animals with cysts in 1,403 (2.2%) cattle originating from various locations in Santa Cruz [40]. Interestingly, the area bordering Brazil showed the highest prevalence (3%) with all cysts being infertile [40,77]. Three years later, Camacho [78] reported a prevalence of 0.2% in 3,330 cattle with the highest prevalence in Terebinto at 6.3%. Then, Cadena [41] investigated the presence of CE in pigs in illegal abattoirs in Santa Cruz and La Paz and also at the official slaughterhouse in La Paz. From 820 animals, CE was found only in 1 animal in Santa Cruz (0.1%), while in La Paz, CE was found in 21 out of 1,450 animals (1.4%). Another investigation, carried out in 1985, in the official abattoir in Santa Cruz de la Sierra found 2.2% of infection after inspecting 504 cattle from Santa Cruz and Beni [79]. In 1988, Gonzalez [80] studied the prevalence in cattle including 59,272 animals (58,483 from Bolivia and 789 from Brazil). Cysts were found in 336 out of 47,818 animals examined from Santa Cruz (0.7%), in 6 out of 779 from Tarija (0.8%), in 47 out of 9,874 from Beni (0.5%), no infection was found in 12 animals from Chuquisaca and in animals from Brazil. Interestingly, all cysts reported were infertile. Also, in Santa Cruz, Aponte [81] found 1.3% of the cattle slaughtered to be infected with *E*. *granulosus*.

**Table 4:** Summary of reports of infection with *E*. *granulosus* in livestock species at postmortem examination at abattoir in the different departments and provinces of Bolivia.

Department	Province/Town	# of animals studied	# of positives (%)	Species	Fertility of cysts	Period	Reference
Beni	Moxos	97	1 (1)	Cattle	No	1969	[40]
Beni	ND	9,874	47 (0.5)	Cattle	No	1988 (7 months)	[80]
Beni	ND	895	13 (1.4)	Cattle	No	2001 (2 months)	[82]
Beni and Santa Cruz	ND	504	11 (2.2)	Cattle	No	1985 (6 months)	[79]
Santa Cruz	Chiquitos/San José Chiquitos	334	7 (2.1)	Cattle	ND	1969	[40]
	Ñuflo de Chavez/Tesoro	140	2 (1.4)				
	Angel Sandoval/San Matias	114	2 (1.7)				
	Velasco/NA	106	1 (0.9)				
	Warnes/Warnes	74	1 (1.3)				
	Santiesteban/La Esperanza	45	0				
	Border with Brazil	590	18 (3.0)				
	**Total**	**1,403**	**31 (2.2)**				
Santa Cruz	German Busch/Puerto Suarez	418	2 (0.5)	Cattle	No	1969 (3 months)	[78]
	Chiquitos/San José Chiquitos	549	1 (0.2)				
	Ñuflo de Chávez/San Javier	191	1 (0.5)				
	Angel Sandoval/San Matias	131	1 (0.7)				
	Andres Ibañez/Terebinto	16	1 (6.3)				
	Several other sites	2,025	0				
	**Total**	**3,330**	**6 (0.2)**				
Santa Cruz	Cordillera/ND	7,710	105 (1.4)	Cattle	No	1988 (7 months)	[80]
	Velasco/ND	2,098	18 (0.9)				
	Ñuflo de Chavez/ND	10,719	49 (0.5)				
	Angel Sandoval/ND	4,021	33 (0.8)				
	Chiquitos/ND	11,161	91 (0.8)				
	Sara/ND	564	3 (0.5)				
	German Busch/ND	687	3 (0.4)				
	Warnes/ND	1,384	5 (0.4)				
	Andrez Ibañez/ND	8,730	29 (0.3)				
	Santiesteban/ND	497	0				
	Ichilo/ND	204	0				
	Florida/ND	43	0				
	**Total**	**47,818**	**336 (0.7)**				
Santa Cruz	ND	37	1 (2.7)	Cattle	No	1994 (3 months)	[83]
Santa Cruz	ND	10	0	Cattle	No	2001 (2 months)	[82]
Santa Cruz	Chiquitos/Abaroa	95	2 (2.1)	Cattle	No	1990 (4 months)	[81]
	Chiquitos/Corralito	125	1 (0.8)				
	Chiquitos/Ipias	33	4 (1.7)				
	Chiquitos/Suarez Arana	60	1 (1.7)				
	Chiquitos/El Carmen	78	2 (2.6)				
	Chiquitos/Naranjo	36	2 (5.6)				
	Chiquitos/Aguas Calientes	70	0				
	Chiquitos/Palmar de las Islas	120	0				
	Chiquitos/San Lorenzo	90	0				
	Chiquitos/San Pedro	40	0				
	Chiquitos/Sucuara	110	0				
	Chiquitos/Trebol	98	0				
	**Total**	**955**	**12 (1.3)**				
Santa Cruz	ND	78	1 (1.3)	Cattle	ND	2001 (3 months)	[84]
Santa Cruz	Florida/Samaipata	97	1 (1)	Pig	Yes	NA	[41]
	Several other sites	723	0				
	**Total**	**820**	**1 (0.1)**				
Cochabamba	ND	60	0	Cattle	No	2001 (2 months)	[82]
Cochabamba	ND	38	1 (2.6)	Cattle	No	1994 (3 months)	[83]
Chuquisaca	ND	607	3 (0.5)	Cattle	No	1994 (3 months)	[83]
Chuquisaca	ND	155	9 (0.7)	Cattle	ND	2001 (3 months)	[84]
Chuquisaca	Luis Calvo/ND	12	0	Cattle		1988 (7 months)	[80]
Tarija	Gran Chaco/ND	779	6 (0.8)	Cattle	No	1988 (7 months)	[80]
Tarija	Arce/ND	113	3 (2.7)	Cattle	ND	1996 (3 months)	[85]
Tarija	ND	16	6 (37.5)	Cattle	ND	2001 (3 months)	[84]
La Paz	Aroma/Vilaque	ND	11	Cattle	ND	1992	[86]
	Bautista Saavedra/Curva	ND	1				
	Ingavi/Desaguadero	ND	27				
	Ingavi/Lacaya	ND	11				
	Ingavi/Pocoata	ND	14				
	Ingavi/Taraco	ND	1				
	Los Andes/Batallas	ND	92				
	Los Andes/Palcoco	ND	27				
	Omasuyos/Achacachi	ND	82				
	Omasuyos/Huatajata	ND	2				
	**Total**		**268**				
La Paz	Aroma/Lahuachaca	20	0	Cattle	No	2001 (2 months)	[82]
	Aroma/Palcoco	430	69 (16)				
	Aroma/Patacamaya	182	12 (6.6)				
	Camacho/Puerto Acosta	30	0				
	Ingavi/Desaguadero	41	3 (7.3)				
	Ingavi/Guaqui	20	0				
	Ingavi/Pocota	274	11 (4.0)				
	Ingavi/Tiahuanacu	25	0				
	Larecaja/Vilaque	189	26 (13.8)				
	Los Andes/Lacaya	250	15 (6.0)				
	Los Andes/Batallas	437	34 (7.8)				
	Omasuyos/Achacachi	256	10 (3.9)				
	**Total**	**2,154**	**180 (8.3)**				
La Paz	Altiplano	14,493	360 (2.5)	Cattle	ND	2010 (3 months)	[87]
La Paz	Los Andes/Batallas	245	5 (2.0)	Pig	Yes	NA	[41]
	Camacho/Ancoraimes	125	3 (2.4)				
	Ingavi/Ticuyo	250	4 (1.5)				
	Murillo/slaughterhouse	470	6 (1.2)				
	Omasuyos/Huatajata	360	3 (0.8)				
	**Total**	**1,450**	**21 (1.4)**				
La Paz	Los Andes/Batallas	2,047	38 (1.9)	Sheep	45%	1998–1999 (3 months)	[88]
Oruro	Sajama/Collana	481	77 (16.0)	Llama	ND	2019 (2 months)	[89]
	Sajama/Jilanaca	138	29 (21.0)				
	Sajama/Jila Pumiri	171	46 (26.9)				
	Sajama/Jacha Salli	206	48 (23.3)				
	Sajama/Sullca Pumiri	134	31 (23.1)				
	Sajama/Sullca Salli	126	32 (25.4)				
	**Total**	**1,256**	**263 (20.9)**				
Potosí	ND	17	0	Cattle		1994 (3 months)	[83]
Potosí	Sud Chichas/ND	183	22 (12.0)	Cattle	ND	1998 (3 months)	[85]
	Omiste/ND	16	1 (6.3)				
	Nor Chichas/ND	20	0				
	Cercado/ND	18	0				
	**Total**	**237**	**23 (9.7)**				
Potosí	Saavedra/Betanzos	991	33 (3.3)	Cattle	ND	2001 (3 months)	[84]

ND, no data.

The only study performed in Sucre included 699 cattle from 3 departments from which only 5 animals (0.7%) were infected with *E*. *granulosus*, including 1/37 from Santa Cruz (2.7%), 1/38 from Cochabamba (2.6%), and 3/607 from Chuquisaca (0.5%). At the same time, no infection was recorded in 17 animals from Potosí [83]. The only work investigating CE prevalence in sheep in the whole country described 38 out of 2,047 sheep (1.9%) positives from Batallas near the cities of La Paz and El Alto. According to the study, 45% of the sheep’s cysts were unfertile [88]. Prevalences in cattle between 0% and 9.7% have been reported in Potosí [83,85]. Unfortunately, there is no published work referring to CE in goats in Bolivia. However, infected goats are found in the valleys in the south of the country.

Finally, the most recent description of CE in cattle from different Bolivia Departments was published as a thesis in 2001 by Reinoso [82], reporting a prevalence of 6.2% from 3,119 cattle. The highest prevalence was identified in animals slaughtered in official abattoirs in La Paz (8.3%). Some areas within the La Paz Department showed prevalences as high as 16% in Palcoco, 13.8% in Vilaque, and 7.8% in Batallas. Data from these theses show that the highest prevalences are at the northern Altiplano of the Department of La Paz, where the disease has been described in cattle, sheep, and pigs [41,82,88]. Unfortunately, only one of the theses included CE reports in sheep, which is the most important intermediate host for *E*. *granulosus s*.*s*. Finally, in the most recent survey of the prevalence of *E*. *granulosus* in animals in 2019, Calle [89] described 20.9% of infection in llamas in Oruro (263 out of 1,256 animals). This high level of infection suggests that llamas could play an essential role in transmitting *E*. *granulosus* in some areas of Bolivia. It remains unknown if *E*. *granulosus s*.*s*. or another species of *E*. *granulosus s*.*l*. complex is responsible for the infection in llamas in Oruro.

### Genetic characterisation of *E*. *granulosus s*.*l*. in Bolivia

*E*. *granulosus s*.*s*. was described for the first time in Bolivia in a publication by Kamenetzky and colleagues [90] from a human sample. No information was given by the authors about the exact origin of the sample. The second characterisation of a human CE sample was recently published in a case report of a 3-year-old girl from a rural area near La Paz and El Alto (discussed in the case reports section) [25]. Finally, Ali and colleagues [8] reported the presence of *E*. *granulosus s*.*s*. in 30 cysts (from La Paz, Cochabamba, and Beni) from sheep, cattle, and humans, *E*. *ortleppi* (G5) in 5 fertile cysts from cattle (from La Paz and Cochabamba) and *E*. *intermedius* (G7) in 3 fertile cysts from pigs (from Santa Cruz). Additionally, *E*. *granulosus s*.*s*. and *E*. *ortleppi* were found in dog faecal samples collected in the La Paz Department.

### Control of *E*. *granulosus* in Bolivia

An attempt to establish a national control programme for CE in Bolivia was proposed and mentioned in the First National Seminar for Control and Surveillance of Zoonoses in 1989 [91]. The plan was intended to last for 4 years; however, it was not implemented. A pilot study for diagnostic in humans and dogs was developed in Tupiza in collaboration with the “Southern cone sub regional initiative for control and surveillance of cystic echinococcosis” between 2009 and 2010. The results are shown in the “Ultrasound diagnosis, treatment, and patient follow-up” section of this review. Several recommendations and training in ultrasound were part of this pilot study [92]. Only a single dog deworming campaign was carried out [70]. Recently, the Ministry of Health established a guide for surveillance and prevention of *E*. *granulosus* and *Fasciola hepatica* [93].

On the other hand, the National Zoonosis Program of the Ministry of Health is limited to rabies vaccination of dogs, without activities against CE. Human CE notification is not compulsory, and the infected viscera from livestock are confiscated at the municipal abattoirs. Staff at the National Service of Agricultural Health and Food Safety (SENASAG) is notified, but not control measures are implemented. The sanitary inspection is limited to bovines in municipal abattoirs located in cities. The uncontrolled clandestine slaughter, mainly of pigs and sheep and South American camelids, persists even in communities close to large cities.

### Epidemiological analysis of *E*. *granulosus* in Bolivia

Data reviewed here demonstrate that this zoonotic disease has been historically reported in 8 out of 9 Departments of this country except for Pando. The livestock population in Bolivia includes 9,304,572 bovines, 5,382,778 sheep, 2,941,827 pigs, 1,454,923 goats, and around 1 million South American camelids [94]. Large-scale intensive livestock rearing, specialised in cattle production, is restricted to the lowlands of the Departments of Beni, Santa Cruz, and Pando in the northeast of Bolivia. In contrast, in La Paz and other Departments, farmers have a small number of animals frequently mixing different species (bovines, ovine, pigs, and camelids). This happens because of the shortage of forage due to the Altiplano’s bioclimatic conditions [95]. Livestock rearing is usually combined with other agricultural activities; supervising livestock is frequently the children’s responsibility.

Considering the available data, we propose the analysis of the CE in 3 ecological levels in Bolivia: Altiplano (La Paz, Oruro, and Potosí), subtropical valleys (Cochabamba, Tarija, and Chuquisaca), and tropical lowlands from the Amazon (Beni, Pando, and Santa Cruz). In the case of the Altiplano, La Paz is the department that reported the majority of the cases and more retrospective studies on CE (see Tables 1 and 2). Unfortunately, the cases’ actual origin is not available in several reports, and the majority of reports are from hospitals in big cities where surgeries were performed (passive detection). Cattle, sheep, and pigs are common in this area, principally in the ecoregion named “wet puna.” The raising of South American camelids is scarce in this region. In our experience, the Northern Bolivian Altiplano at La Paz Department is one of the most endemic areas for CE in Bolivia. The transmission is low in certain valleys with few human cases reviewed here (Tables 1 and 2), while animal infection reached up to 37.5% in Tarija, for example (Table 4).

In contrast, 44 human cases have been reported in Chuquisaca, including the first autochthonous case from Bolivia; however, a low prevalence in livestock has been found in this department (Table 3). More precise information is available from Potosí’s valleys. Only 1 epidemiological study involving several communities in Tupiza reveals the prevalence of CE between 2.1% and 13.3% in humans using ultrasound examination [62] and also the infection in dogs. In the case of the lowlands, livestock is found in large-scale intensive systems primarily cattle. Low CE prevalence has been described for this area for livestock, while no reports of CE in humans have been described in recent years. However, the identification of *E*. *intermedius* G7 in pigs from Santa Cruz [8] warrants further investigation in this Department. Previously, *Echinococcus vogeli* was identified in Santa Cruz infecting *Cuniculus paca* [96].

## Conclusions

The available information on CE in Bolivia does not allow a comprehensive countrywide estimation of the disease’s epidemiological situation and burden. However, data summarised here show that CE is present in 8 out of 9 Departments in Bolivia. CE became more commonly diagnosed in the last 50 years. The maximum levels of infection recorded in livestock in different species from Bolivia also show that the parasite is widely spread. Studies performed in dogs showed that the parasite is actively transmitted in areas near major population centres like La Paz and El Alto.

Furthermore, the recent detection of 3 different species of *E*. *granulosus s*.*l*. in Bolivia warrants further epidemiological investigations. Future baseline epidemiological data, including studies in dogs and intermediate hosts and a systematic collection of human cases, are necessary. Data acquired will form the basis for the discussion and implementation of national control programmes. Public health education will be essential due to the lack of knowledge of the disease in the Bolivian population. Promote the interest related to CE and the sensibility of the national and departmental authorities to assume influential and integral responsibilities in the context of “One Health” is an objective of this work. The management based on standardised protocols of the human CE in hospitals could clarify the disease’s epidemiological situation in this country. Finally, further studies investigating other related species present in some South American countries including Bolivia, as *E*. *vogeli* or *Echinococcus oligarthrus*, are also indicated.

**Key Learning Points**

- There is no systematic collection of data for infection in humans, livestock, or dogs caused by *E*. *granulosus s*. *l*. in Bolivia.
- Human CE has been diagnosed in Bolivia since the first half of the 19th century, and cases have increased since the 1970s.
- Current evidence suggests an unequal geographic distribution of *E*. *granulosus s*. *l*. in Bolivia with higher infection in the departments of La Paz and Potosí.
- Llamas could play an essential role in maintaining the cycle of the parasite in the Bolivian Altiplano.
- There is an urgent need for studies to understand the epidemiology of CE in all departments of Bolivia.

**Top Five Papers**

1. Villena E. Fortalecimiento cooperación técnica para mejorar el control de la hidatidosis en Bolivia. Proyecto de Fortalecimiento de la Cooperación Técnica entre Uruguay y Bolivia para mejorar el control de la Hidatidosis en Bolivia. Tupiza: WHO/PAHO2011.
2. Ali V, Martinez E, Duran P, Seláez MA, Barragan M, Nogales P, et al. *Echinococcus granulosus sensu stricto*, *Echinococcus ortleppi* and *Echinococcus intermedius* (G7) are present in Bolivia. Parasitology. 2020;147(9):949–56.
3. Sarmiento G. Estudio retrospectivo de la frecuencia de hidatidosis en los centros hospitalarios especializados y de diagnóstico de la ciudad de La Paz Bolivia. Universidad Mayor de San Andrés; 1998.
4. Tamayo L, Pacheco R, Fernandez R, Chungara J. Hidatidosis. Experiencia institucional. Rev Soc Bol Ped. 2004;43(3):149–54.
5. Calle R. Determinación de la prevalencia post mortem de *Echinococcus granulosus* (hidatidosis) en llamas (*Lama glama*) en seis diferentes comunidades del municipio de Turco del departamento de Oruro. Universidad Mayor de San Andrés 2019.
